# Effects of SGLT2 Inhibitors beyond Glycemic Control—Focus on Myocardial SGLT1

**DOI:** 10.3390/ijms22189852

**Published:** 2021-09-12

**Authors:** Alex Ali Sayour, Mihály Ruppert, Attila Oláh, Kálmán Benke, Bálint András Barta, Eszter Zsáry, Béla Merkely, Tamás Radovits

**Affiliations:** Heart and Vascular Center, Department of Cardiology, Semmelweis University, Városmajor str. 68, H-1122 Budapest, Hungary; ruppert.mihaly@med.semmelweis-univ.hu (M.R.); olah.attila@med.semmelweis-univ.hu (A.O.); benke.kalman@med.semmelweis-univ.hu (K.B.); barta.balint_andras@med.semmelweis-univ.hu (B.A.B.); eszterzsary@gmail.com (E.Z.); merkely.bela@med.semmelweis-univ.hu (B.M.); radovits.tamas@med.semmelweis-univ.hu (T.R.)

**Keywords:** sodium–glucose cotransporter 1, SGLT2 inhibitor, dual SGLT1/2 inhibitor, heart failure, myocardial ischemia, empagliflozin, sotagliflozin

## Abstract

Selective sodium–glucose cotransporter 2 (SGLT2) inhibitors reduced the risk of hospitalization for heart failure in patients with or without type 2 diabetes (T2DM) in large-scale clinical trials. The exact mechanism of action is currently unclear. The dual SGLT1/2 inhibitor sotagliflozin not only reduced hospitalization for HF in patients with T2DM, but also lowered the risk of myocardial infarction and stroke, suggesting a possible additional benefit related to SGLT1 inhibition. In fact, several preclinical studies suggest that SGLT1 plays an important role in cardiac pathophysiological processes. In this review, our aim is to establish the clinical significance of myocardial SGLT1 inhibition through reviewing basic research studies in the context of SGLT2 inhibitor trials.

## 1. Introduction

As the incidence of type 2 diabetes mellitus (T2DM) is steadily increasing [1], sodium–glucose cotransporter 2 (SGLT2) inhibitors have been developed as a novel class of antihyperglycemic agents. To date, several large cardiovascular outcome trials in high-risk T2DM patients have been conducted with selective SGLT2 inhibitors [2,3,4,5,6] and one with the dual SGLT1/2 inhibitor sotagliflozin [7]. Not only these medications have proven to be safe, but they have shown robust salutary cardiorenal protection as a class effect, with currently unclear mechanism of action.

## 2. The Rationale behind Pharmacological SGLT2 and SGLT1 Inhibition

Selective SGLT2 inhibitors and the dual SGLT1/2 inhibitor sotagliflozin were originally designed to aid glucose control in patients with diabetes mellitus. This pharmacological action is based on the blockade of SGLT2 in the kidney. Renal SGLT2 is a high-capacity glucose transporter that uses one sodium ion per glucose molecule to transport glucose into the intracellular space [8]. It is located in the brush border of the proximal convoluted tubule (S1/S2 segment) of kidney nephrons accounting for the reabsorption of the majority of glucose (~97%) under normoglycemic conditions [9]. On the contrary, the low-capacity SGLT1 is abundant in the brush border of the distal part (S3 segment) of the proximal convoluted tubule, and accounts for the reabsorption of remnant glucose (~3%) [10]. SGLT1 uses two sodium ions per glucose molecule [8], making it more energy-consuming than SGLT2.

Since SGLT2 is upregulated in the kidneys of humans (and rodents) with diabetes [11] and at the same time accounts for virtually all of the glucose reabsorption in the kidney, it is well-established that pharmacological inhibition of renal SGLT2 results in glucosuria and reduced serum glucose levels. However, selective SGLT2 inhibition is associated with 40–50% glucose reabsorption, as it unleashes the transport capacity of the distal SGLT1 [10,12], which compensates for the loss of SGLT2 activity in the kidney to some extent. Interestingly, the dual SGLT1/2 inhibitor sotagliflozin has a glucosuric effect similar to that of selective SGLT2 inhibitors [13], but it was originally designed to block intestinal SGLT1, where SGLT1 shows the highest expression in the body, accounting for the vast majority of glucose absorption [14]. Herein, intestinal SGLT1 inhibition results in delayed glucose uptake and release of glucagon-like peptide 1 (GLP-1), which might contribute to improved glycemic control [15].

## 3. Cardiorenal Benefits of Pharmacological SGLT2 and Dual SGLT1/2 Inhibition in Patients with and without Type 2 Diabetes Mellitus

Large cardiovascular outcome trials have been carried out with the following selective SGLT2 inhibitors: empagliflozin [2], canagliflozin [3,5], dapagliflozin [4], and ertugliflozin [6] (Table 1). These trials enrolled high-risk T2DM patients and showed that SGLT2 inhibitors only slightly reduce major adverse cardiovascular events (MACE), but all trials reported a highly significant ~32% relative reduction in risk of hospitalization for HF with no heterogeneity [16]. It is worth noting that the event curves of hospitalization for HF separated very quickly in all trials, being significantly lower in the SGLT2 inhibitor arm already after 1 month of treatment in some cases. The SCORED trial further reinforced the consistency of these salutary effects by showing a similarly large (33%) relative risk reduction in hospitalization for HF and urgent visits for HF with the dual SGLT1/2 inhibitor sotagliflozin [7]. However, unlike individual SGLT2 inhibitors, sotagliflozin also significantly reduced the risk of myocardial infarction and stroke, respectively [7]. Therefore, it seems that, in patients with T2DM, additional SGLT1 inhibition on top of SGLT2 blockade might be effective against macrovascular endpoints.

The first dedicated HF trial with the SGLT2 inhibitor dapagliflozin (DAPA-HF) [17] showed that treatment resulted in significantly reduced risk of first worsening HF event and death from cardiovascular causes in patients with reduced ejection fraction, irrespective of the presence of T2DM. A similar reduction in the composite endpoint was documented in another dedicated HF trial (EMPEROR-Reduced) [18] in patients with reduced ejection fraction, again, independent of the presence of diabetes. Therefore, the salutary cardiovascular effects of selective SGLT2 inhibitors are not confined to diabetic conditions in patients with HF. The dual SGLT1/2 inhibitor sotagliflozin was also tested in a dedicated HF trial (SOLOIST-WHF) [19]; however, exclusively, patients with T2DM and recent hospitalization for worsening HF were enrolled. The primary endpoint (composite of cardiovascular death, hospitalizations, and urgent visits for HF) was sharply reduced by 33% in the sotagliflozin arm, as compared with placebo [19]. Furthermore, the SOLOIST-WHF was the first dedicated HF trial to document a significant subgroup effect regarding the reduction in the risk of the composite outcome in patients with HF and preserved ejection fraction [19]. The EMPEROR-Preserved trial reinforced these findings in patients with HF and preserved ejection fraction with or without T2DM [20]. Therefore, SGLT2 inhibitors and the dual SGLT1/2 inhibitor sotagliflozin are no longer simply antihyperglycemic agents, but represent a new class of HF medications (Table 2).

## 4. Proposed Mechanisms of Cardiovascular Protective Effects of SGLT2 Inhibitors—Why Myocardial SGLT1 Matters

**Figure 1:**
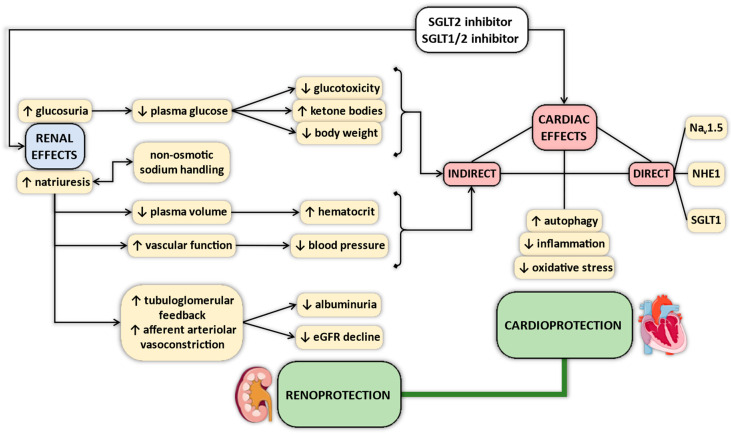
Summary of proposed pleiotropic effects of sodium–glucose cotransporter 2 (SGLT2) inhibitors. eGFR = estimated glomerular filtration rate; SGLT1 = sodium–glucose cotransporter 1; SGLT2 = sodium–glucose cotransporter 2; NHE1 = Na^+^/H^+^-exchanger 1.

Several mechanisms have been proposed that could explain the clinically observed salutary cardiovascular effects of SGLT2 inhibitors [21,22,23,24,25,26,27,28,29,30,31] (Figure 1). Currently, the exact mechanism is unclear, but there is good reason to believe in pleiotropic actions, which might differ in importance. For example, antihyperglycemic actions might not play a key role in non-diabetic patients with HF, in whom SGLT2 inhibitors are equally effective [17,18,32]. Osmotic and natriuretic effects might also be less dominant, since SGLT2 inhibitors have little effect on markers of fluid volume overload in patients with HF [17,18,33], and beneficial clinical outcomes are equivalent in HF patients irrespective of whether or not they experienced recent manifestation of volume overload [34]. Salutary renal actions of SGLT2 inhibitors in addition to diuretic effects have also been proposed, independent of diabetic state [28].

There is a growing number of studies suggesting that SGLT2 inhibitors exert direct cardioprotective effects. However, SGLT2 is not expressed in murine and human hearts to a relevant extent [35,36,37,38,39,40,41]. Few studies have reported the possible membrane transporter in cardiomyocytes that might convey the signal of SGLT2 inhibitors into the intracellular space. The Na^+^/H^+^-exchanger 1 (NHE1) has recently been identified as a potential membrane transporter that is blocked by selective SGLT2 inhibitors in healthy rabbit, rat, and mouse cardiomyocytes [42,43,44]. This effect seems to be vastly different from that of the NHE1 inhibitor cariporide under pathological conditions [45], whereas others reported no substantial effect of SGLT2 inhibitors on NHE1 activity in cardiomyocytes [46]. Finally, another study identified the Na_v_1.5 channel in cardiomyocytes as a potential target for SGLT2 inhibitors [47].

Interestingly, a recent study using docking analysis found that the most selective SGLT2 inhibitor empagliflozin shows a relatively high binding affinity towards SGLT1, with much less affinity towards NHE1 [48]. Given the wide range of the selectivity of SGLT2 inhibitors for SGLT2 over SGLT1 (ranging from 260 to 2700-fold [49,50]) and the dual SGLT1/2 inhibitory property of sotagliflozin, it is reasonable to speculate that less selective agents could bind to myocardial SGLT1 with higher potency, exerting direct cardiac actions. Importantly, SGLT1 is highly expressed in the myocardium [35,36,37,38,39,40,41,51] and its expression is altered in disease states. A recent study found that clinically relevant plasma concentrations of the least selective SGLT2 inhibitor canagliflozin reduced nitro-oxidative stress in human cardiomyocytes, which was SGLT1-dependent [35]. Interestingly, these findings could not be recapitulated with the highly selective SGLT2 inhibitor empagliflozin [35], suggesting that the degree of selectivity for SGLT2 over SGLT1 has important clinical implications.

A pivotal study established the relevance of SGLT1 inhibition on the population level, as well. Seidelmann and colleagues [52] found that persons with alterations in the SLC5A1 gene—resulting in production of functionally damaged SGLT1—are at substantially lower risk of developing T2DM and HF (23% and 30% relative risk reductions, respectively). All-cause death was significantly reduced in a 25-year follow up period as compared with genetically unaffected controls [52]. On the contrary, polymorphisms in the SLC5A2 gene (encoding SGLT2) were found to be associated with very small reductions in the incidence of HF and T2DM (both <3% relative risk reduction), whereas all-cause mortality was unaffected [53].

Based on the above, it cannot be ruled out that SGLT1 inhibition could contribute to the salutary cardiorenal effects of SGLT2 inhibitors. In the following sections, we highlight how SGLT1 contributes to pathophysiological processes in the heart, so that the beneficial cardiac effects of myocardial SGLT1 inhibition could be appreciated.

## 5. Changes in Expression of SGLT1 in Various Myocardial Disease States

Several studies documented that humans with HF exhibit increased LV SGLT1 mRNA or protein expression as compared with non-failing controls, including those with dilated cardiomyopathy (DCM) [41], ischemic cardiomyopathy (ICM) [39,41,51], hypertrophic cardiomyopathy (HCM) [39], and also those with T2DM [41,51,54], or mixed cohorts of these HF etiologies [54]. Some studies found no significant difference in LV SGLT1 expression in patients with HCM [41], or DCM [40,51], or ICM [40] compared with non-failing controls. Interestingly, in patients undergoing LV assist device (LVAD) implantation, apical SGLT1 mRNA expression increased after weaning compared to baseline [51], hinting a compensatory role in functional recovery. However, periprocedural ischemia and local inflammation in the apical region in conjunction with the LVAD inflow cannula might also explain increased SGLT1 expression. Table 3 summarizes these findings in humans.

Data are scarce regarding the possible regulators of myocardial SGLT1 expression in human hearts. A previous study identified that in patients with HCM or ICM, SGLT1 expression increased in conjunction with the activating phosphorylation of adenosine monophosphate-activated protein kinase (AMPK) and extracellular signal-regulated protein kinase 1/2 (ERK1/2) [39]. However, in a relatively large number of patients with end-stage HF, we found a significant upregulation of LV SGLT1 mRNA and protein expression compared with controls which was accompanied by unaltered AMPK phosphorylation, whereas ERK1/2 activation was significantly lower [41].

In line with data on human hearts, myocardial SGLT1 mRNA or protein expression was found to be upregulated in non-diabetic small animal models of acute myocardial ischemia–reperfusion injury [55] or ischemic preconditioning [56], permanent LAD ligation (model of ICM) [51,57,58], and hemodynamic-overload induced HF [59,60], as well as in models of metabolic syndrome and T2DM [51,54,61,62,63,64]. Interestingly, SGLT1 was downregulated in mice with streptozotocin-induced type 1 diabetes mellitus (T1DM) [51]. Some preclinical studies showed no significant alteration in myocardial SGLT1 expression in hearts of mice with metabolic syndrome [65], or following permanent LAD ligation [66], or acute ex vivo ischemia–reperfusion injury [65,67]. Table 4 summarizes these findings in small animal models.

In mice, AMPK and ERK1/2 were found to be responsible for upregulating myocardial SGLT1 during acute ischemia–reperfusion injury [55]. In a genetic model of HF, overactivation of AMPK resulted in substantial upregulation of myocardial SGLT1 [68,69]. In rats with chronic pressure overload-induced HF, we found increased LV SGLT1 expression with preserved ERK1/2 phosphorylation [60]. On the contrary, HF due to chronic volume overload was associated with comparable upregulation of SGLT1 but significantly lower ERK1/2 phosphorylation, whereas AMPK activity was diminished in both models [60]. Therefore, in chronic HF, other mediators might contribute to maintaining increased SGLT1 expression, although a negative association between ERK1/2 activation and SGLT1 expression has been noted in primary cultured rabbit renal proximal tubule cells [70,71] and in humans with end-stage HF [41].

## 6. Localization of Myocardial SGLT1

Zhou and colleagues [36] were the first to specifically show that SGLT1 mRNA was present in human cardiomyocytes originating from normal heart tissues, using in situ hybridization technique. In fact, the abundance of SGLT1 mRNA levels in the myocardium was second only to the small intestine [36]. Later, it was found that capillaries of rat hearts also express SGLT1, as do primary cultured coronary endothelial cells [72] and human cardiac fibroblasts [73]. Banerjee and colleagues [51] performed SGLT1 immunofluorescent staining on cardiomyocytes and speculated that it localized to the sarcolemma. A similar staining pattern was reported in another study in normal and infarcted rat heart samples [57]. Immunoblotting of the membrane fraction of murine cardiac tissue revealed high expression of SGLT1 which was absent from the cytosolic fraction [51,56]. In fact, cardiomyocytes with increased expression of SGLT1 exhibit similar distribution of SGLT1 to those of normal cardiomyocytes, being co-localized with the sarcolemma marker Na^+^-K^+^-ATPase [68]. In human hearts, a similar immunofluorescent staining pattern of SGLT1 was documented [39]. Therefore, it is unlikely that a significant cytosolic pool contributes to SGLT1 expression, even if it is upregulated.

However, Vrhovac and colleagues [74] found that SGLT1 did not co-localize with Na^+^-K^+^-ATPase in the human heart, instead it co-localized with aquaporin-1, a marker of capillaries. The authors postulated that SGLT1 expression in the heart is confined to the capillaries of the myocardium [74]. In contrast, we showed that SGLT1 co-localized with Na^+^-K^+^-ATPase in the membrane of cardiomyocytes in LV sections from patients with end-stage HF [41]. Furthermore, SGLT1 immunohistochemical staining revealed a diffuse distribution in cardiomyocytes, whereas fibrotic tissue and adipocytes were not meaningfully stained [41]. This is in line with the study of Kashiwagi et al. [67] showing a diffuse positive staining of SGLT1 in tissues obtained from all four chambers of human autopsied hearts. A similar pattern confined to cardiomyocytes was reported in healthy and diabetic rat hearts [64]. On the single cell level, studies confirmed that cardiomyocytes isolated from normal and diabetic hearts [54], and from HL-1 cardiac cell line (murine atrial cardiomyocytes) [55] express high levels of SGLT1 in the membrane. Consequently, the totality of evidence support that cardiomyocytes are the dominant cell types in the heart that express SGLT1, nonetheless, the microvasculature [72,74] and fibroblasts [73] are also involved.

## 7. Role of Myocardial SGLT1 in Glucose Uptake

Neither global knock out [75,76] nor cardiomyocyte-specific knock down [55] of SGLT1 alters the baseline glucose uptake on the cardiomyocyte or myocardial level. Furthermore, no specific basal phenotype has been noted in these mice, as heart weight, myocardial structure, and LV function are unchanged [38,55,69,76]. Similarly, pharmacological SGLT1 inhibition with the non-specific SGLT inhibitor phlorizin does not alter baseline glucose uptake in healthy hearts or cardiomyocytes, whereas baseline cardiac function is unaffected [54,65,67,68]. However, phlorizin has been found to strongly inhibit GLUTs in cardiomyocytes [76], rendering it unreliable to study the relevance of SGLT1 inhibition in the heart.

Unexpectedly, the increase in glucose uptake in cardiomyocytes from mice with global SGLT1 knockout was similar to that of wild-type controls during insulin stimulation or hyperglycemia [76]. Furthermore, cardiomyocyte-specific SGLT1 knock down did not alter the increase in myocardial glucose uptake in relation to ischemia–reperfusion injury [55]. Nonetheless, cardiomyocyte-specific overexpression of SGLT1 for 10 weeks in mice results in substantial accumulation of glycogen content in the myocardium (possibly through increased glucose uptake) which can be reversed by SGLT1 knock down thereafter [69]. Finally, overexpression of SGLT1 through constitutively active AMPK also results in glycogen accumulation, which is prevented by suppression of AMPK activity and subsequent normalization of SGLT1 expression [69]. Therefore, chronic upregulation of SGLT1 might indeed be associated with increased glucose uptake in cardiomyocytes, unlike in acute phases. Phlorizin was reported to inhibit glucose uptake in cardiomyocytes or myocardium under various stimulated conditions, including acute ex vivo ischemia–reperfusion injury [65,67], T2DM [54], and insulin or leptin stimulation [51]. However, the non-specificity of phlorizin again limits the interpretation of these results.

## 8. Role of Myocardial SGLT1 under Diabetic Conditions

**Figure 2:**
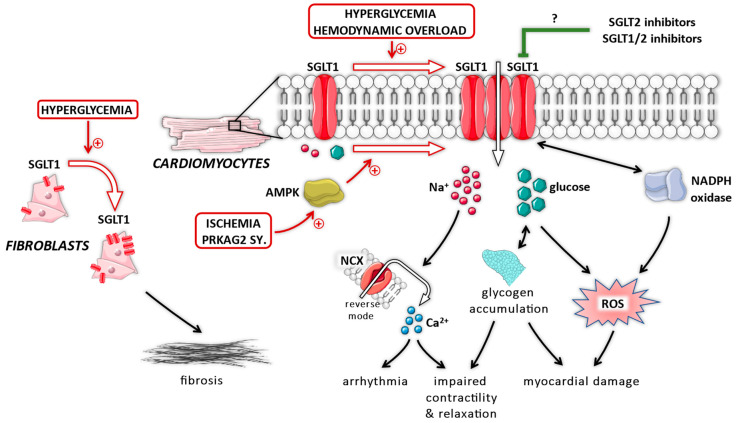
The putative role of myocardial sodium–glucose cotransporter 1 (SGLT1) in pathophysiological processes. AMPK = adenosine monophosphate-activated protein kinase; NADPH = nicotinamide adenine dinucleotide phosphate; NCX = Na^+^/Ca^2+^ exchanger; ROS = reactive oxygen species; SGLT1 = sodium–glucose cotransporter 1; SGLT2 = sodium–glucose cotransporter 2; SY = syndrome.

Several studies documented that SGLT1 plays an important pathophysiological role in the myocardium under diabetic conditions (Figure 2), as it might translate extracellular glucose overload into intracellular nitro-oxidative stress. In adult rat cardiomyocytes, exposure to high-glucose containing medium facilitates increased reactive oxygen species (ROS) production mediated by nicotinamide adenine dinucleotide phosphate (NADPH) oxidase 2 isoform (Nox2) activation, which could be blocked by the SGLT inhibitor phlorizin, but not by the GLUT inhibitor phloretin [77]. Even though phlorizin can block GLUTs, phloretin could not reduce ROS production in relation to glucose overload, whereas SGLT2 is not expressed in cardiomyocytes. Therefore, SGLT1 might be involved in facilitating intracellular nitro-oxidative stress under diabetic conditions. Indeed, in mice with T2DM presenting with increased expression of LV SGLT1, knockdown of SGLT1 reduced myocardial nitro-oxidative stress and inflammation, and resulted in preservation of LV systolic and diastolic function [62,63].

Importantly, SGLT1 transfers two sodium ions down the electrochemical gradient to bring one glucose molecule inside the cell. Therefore, chronic upregulation of SGLT1 in cardiomyocytes might not only result in intracellular glycogen accumulation, but also intracellular Na^+^ overload. The latter is a well-known phenomenon in HF [78] and contributes to its pathophysiology [79]. Lambert and colleagues [54] found that LV SGLT1 protein expression was significantly upregulated in HF patients, but obesity and T2DM were associated with further increases. Cardiomyocytes originating from rats with T2DM with increased SGLT1 expression showed higher intracellular Na^+^ levels compared with non-diabetic controls, which could be further elevated during electric stimulation [54]. However, phlorizin or glucose-free medium greatly prevented Na^+^ uptake (while the Na^+^/K^+^-ATPase was blocked by ouabain) in cardiomyocytes originating from these diabetic rats, but not in those from healthy rats [54]. The fact that glucose-free solution also prevented Na^+^ uptake suggests a causal role of SGLT1, as the transporter uses the Na^+^ gradient to bring glucose into cells, unlike GLUTs. When extracellular glucose is absent (and the Na^+^/K^+^-ATPase is blocked by ouabain), SGLT1 is inactive and, therefore, no Na^+^ is transported into the intracellular space via this transporter, preventing intracellular Na^+^ accumulation.

Besides cardiomyocytes, cardiac fibroblasts also express SGLT1 which might play an important role in the development of cardiac fibrosis. In human cardiac fibroblasts, high-glucose medium increased SGLT1 and matrix metalloproteinase 2 expression which was blocked by phlorizin [73]. This was also confirmed in rat cardiac fibroblasts in vitro, whereas in a rat model of T2DM, LV SGLT1 expression was upregulated together with increased expression of collagen I and III, explaining the higher levels of cardiac interstitial fibrosis [64]. Overexpression of SGLT1 in rat cardiac fibroblasts in vitro is sufficient to promote collagen release, which could be prevented by knock down of SGLT1 [64]. Accordingly, in rats with T2DM, knock down of SGLT1 in vivo significantly downregulated the expression of profibrotic factors, and prevented the accumulation of interstitial fibrosis [64].

## 9. Role of Myocardial SGLT1 under Non-Diabetic Conditions

Several studies documented that SGLT1 contributes to cardiac perturbations under non-diabetic conditions (Figure 2). A recent study confirmed a pivotal relationship between myocardial SGLT1 and the level of nitro-oxidative stress. Specifically, in atrial samples from mostly non-diabetic patients with ischemic CM, higher SGLT1 expression was associated with increased NADPH oxidase-related ROS production, pro-fibrotic, and pro-inflammatory gene expression [35]. In these samples, NADPH oxidase activation and subsequent oxidative damage was suppressed by the least selective SGLT2 inhibitor canagliflozin, but not by the most selective empagliflozin, and this effect seemed to be dependent on SGLT1 [35]. We also reported that canagliflozin blunts oxidative stress in non-diabetic rats with acute myocardial ischemia–reperfusion injury [80]. In line with these, we showed that LV SGLT1 expression is upregulated in non-diabetic rats with HF, irrespective of whether chronic pressure (transverse aortic constriction, TAC) or volume (aortocaval fistula) overload was the underlying pathophysiology [60]. The expression of SGLT1 showed a robust correlation with the extent of myocardial nitro-oxidative stress in rats with HF [60]. Mouse neonatal cardiomyocytes with genetically ablated SGLT1 are resistant to in vitro hypertrophic stimuli, whereas mice with global SGLT1 knockout are protected from the development of pathological LV hypertrophy in response to chronic pressure overload induced by TAC [59]. Compared with wild-type mice, knockout of SGLT1 resulted in preserved LV structure and function, and reduced fibrotic content with suppressed gene expression of profibrotic genes (CTGF, collagen 1), whereas the TAC-induced spike in interleukin-18 expression was prevented [59]. Therefore, SGLT1 might promote myocardial nitro-oxidative stress, inflammation, and fibrosis in response to hemodynamic overload, suggesting an important pathophysiological role in the development of HF. Finally, we have reported that myocardial LV SGLT1 expression correlates positively with LV dilation and dysfunction in patients with HF, independent of age, sex, and body mass index [41]. Subsequently, the level of expression of myocardial SGLT1 might capture the severity of HF.

Myocardial SGLT1 not only contributes to the development of HF, but its conditional cardiomyocyte-specific overexpression for 10 weeks itself is sufficient to evoke LV dilation and systolic dysfunction coupled with increased myocardial glycogen content in mice [69]. However, when SGLT1 was genetically suppressed after this period, LV structure and function returned to normal, and myocardial glycogen and fibrotic content significantly decreased [69]. In a genetic model of HF related to constitutive activation of AMPK (model of *PRKAG2* cardiomyopathy), myocardial glycogen content was increased alongside higher SGLT1 membrane expression [68]. In these mice, knock down of AMPK significantly reduced glucose uptake and myocardial glycogen content by reducing SGLT1 expression [68]. The causal role of SGLT1 in *PRKAG2* cardiomyopathy has been established by evidencing that cardiomyocyte-specific knock down of SGLT1 in this genetic HF model rescued the cardiac phenotype [69].

Apart from HF, SGLT1 has been implicated in the pathophysiology of myocardial ischemia. A recent study showed that knock down of SGLT1 increased cell viability following hypoxia–reoxygenation, and protected against acute myocardial ischemia–reperfusion injury in both in vivo and ex vivo settings, without affecting glucose uptake [55]. As compared with wild-type controls, these mice exhibited smaller infarct sizes with subsequent amelioration in LV function and reduced myocardial nitro-oxidative stress following ischemia–reperfusion [55]. Additionally, the role of SGLT1 in tissue ischemia has been well-documented in the brain. Cerebral ischemia is associated with upregulation of cerebral SGLT1 in mice [81], its knock down reduces infarct size and behavioral abnormalities [82]. While reducing infarct size is not equal to reducing the risk of an ischemic event, it is notable that only the dual SGLT1/2 inhibitor sotagliflozin reduced the risk of myocardial infarction and stroke in patients with T2DM, but not selective SGLT2 inhibitors. Finally, SGLT1 knock out protects against renal ischemia–reperfusion injury [83], whereas SGLT2 knock out does not [84]. These further reinforce the dominant role of SGLT1 over SGLT2 in tissue ischemia.

## 10. Conclusions and Future Directions

There is currently limited evidence that SGLT2 or SGLT1/2 inhibitors significantly interact with myocardial SGLT1, and if they indeed do so, the clinical relevance of this interaction is currently unclear. Nonetheless, SGLT1 plays a pathophysiological role in the heart under various conditions independent of diabetes, including ischemia and HF, whereas individuals with functionally limited SGLT1 are at lower risk of developing HF. Therefore, even if SGLT2 inhibitors do not significantly affect myocardial SGLT1 (which seems to be unlikely), the modulation of this transporter remains a potential target to counteract adverse processes in the heart.

## Figures and Tables

**Table 1:** Summary of cardiovascular outcome trials with sodium–glucose cotransporter 2 (SGLT2) inhibitors and the dual SGLT1/2 inhibitor sotagliflozin in patients with type 2 diabetes mellitus.

Trial Name (Acronym)	Population	Type of SGLT2 Inhibitor (vs. Placebo)	Selectivity for SGLT2 over SGLT1	Primary Outcome	Hazard Ratio (and 95% CI) for Primary Outcome
EMPA-REG OUTCOME (ref: [2])	T2DM + established CVD	empagliflozin	~2700	CV death, nonfatal myocardial infarction, and nonfatal stroke	0.86 (0.74, 0.99) *
CANVAS Program (ref: [3])	T2DM ± CVD (66% established CVD)	canagliflozin	~260	CV death, nonfatal myocardial infarction, and nonfatal stroke	0.86 (0.75, 0.97) *
DECLARE-TIMI 58 (ref: [4])	T2DM ± CVD (41% established CVD)	dapagliflozin	~1200	CV death, nonfatal myocardial infarction, and nonfatal ischemic stroke	0.93 (0.84, 1.03)
CREDENCE (ref: [5])	T2DM + albuminuric CKD (50% established CVD)	canagliflozin	~260	renal composite	0.70 (0.59, 0.82) ***
VERTIS CV (ref: [6])	T2DM + established CVD	ertugliflozin	~2200	CV death, nonfatal myocardial infarction, and nonfatal stroke	0.97 (0.85, 1.11)
SCORED (ref: [7])	T2DM + CKD (89% established CVD)	sotagliflozin	~20	CV death, hospitalization for HF, and urgent visits for HF	0.74 (0.63, 0.88) ***

Asterisks demonstrate the level of significance of the given primary endpoint (* *p* < 0.05, *** *p* < 0.001). Absence of asterisk denotes nonsignificant finding. CI = confidence interval; CKD = chronic kidney disease; CV = cardiovascular; CVD = cardiovascular disease; T2DM = type 2 diabetes mellitus.

**Table 2:** Summary of large-scale dedicated heart failure (HF) trials with sodium–glucose cotransporter 2 (SGLT2) inhibitors and the dual SGLT1/2 inhibitor sotagliflozin.

Trial Name (Acronym)	Population	Type of SGLT2 Inhibitor (vs. Placebo)	Selectivity for SGLT2 over SGLT1	Primary Outcome	Hazard Ratio (and 95% CI) for Primary Outcome
DAPA-HF (ref: [17])	HFrEF ± T2DM	dapagliflozin	~1200	worsening HF and CV death	0.74 (0.65, 0.85) ***
EMPEROR- Reduced (ref: [18])	HFrEF ± T2DM	empagliflozin	~2700	hospitalization for worsening HF and CV death	0.75 (0.65, 0.86) ***
SOLOIST-WHF (ref: [19])	recent hospitalization for HF + T2DM	sotagliflozin	~20	hospitalizations and urgent visits for HF, and CV death	0.67 (0.52, 0.85) ***
EMPEROR- Preserved (ref: [20])	HFpEF ± T2DM	empagliflozin	~2700	hospitalization for HF and CV death	0.79 (0.69, 0.90) ***

Asterisks demonstrate the level of significance of the given primary endpoint (*** *p* < 0.001). CI = confidence interval; CV = cardiovascular; HF = heart failure; HFpEF = heart failure with preserved ejection fraction; HFrEF = heart failure with reduced ejection fraction; T2DM = type 2 diabetes mellitus.

**Table 3:** Summary of studies in humans investigating the expression of myocardial sodium–glucose cotransporter 1 (SGLT1) in various pathological conditions compared with healthy controls.

		SGLT1 Expression				
Condition	Subtype	Ref: [41]	Ref: [39]	Ref: [51]	Ref: [54]	Ref: [40]
HF	hypertrophic CM	**~**	**↑**		(**↑**)	
HF	ischemic CM	**↑**	**↑**	**↑**	(**↑**)	**~**
HF	dilated CM	**↑**		**~**	(**↑**)	**~**
HF	metabolic syndrome/T2DM	**↑**		**↑**	**↑**	
HF	post-LVAD			**↑**		

Upwards arrows denote significantly increased expression of myocardial SGLT1 compared with controls, whereas upwards arrows in parenthesis denote significantly increased expression of myocardial SGLT1 when the heart failure (HF) group constituted a mix of these subtypes. Tildes denote no significant difference compared with controls. CM = cardiomyopathy; HF = heart failure; LVAD = left ventricular assist device; ref = reference; T2DM = type 2 diabetes mellitus.

**Table 4:** Summary of studies in small animals investigating the expression of myocardial sodium–glucose cotransporter 1 (SGLT1) in various pathological conditions compared with healthy controls.

Condition	SGLT1 Expression	Ref:	SGLT1 Expression	Ref:
acute myocardial IRI	↑	[55,56]	~	[65,67]
permanent LAD ligation	↑	[51,57,58]	~	[64]
hemodynamic overload-induced HF	↑	[59,60]		
metabolic syndrome/T2DM	↑	[51,54,61,62,63,64]	~	[65]
T1DM	↓	[51]		

Upwards arrows denote significantly increased expression of myocardial SGLT1 compared with controls. Tildes denote no significant difference compared with controls. HF = heart failure; IRI = ischemia–reperfusion injury; LAD = left anterior descending coronary artery; ref = reference; T1DM = type 1 diabetes mellitus; T2DM = type 2 diabetes mellitus.
